# *Sorghum bicolor INDETERMINATE1* is a conserved primary regulator of flowering

**DOI:** 10.3389/fpls.2023.1304822

**Published:** 2023-12-13

**Authors:** Samuel De Riseis, Junping Chen, Zhanguo Xin, Frank G. Harmon

**Affiliations:** ^1^ Department of Plant & Microbial Biology, University of California, Berkeley, Berkeley, CA, United States; ^2^ Plant Stress and Germplasm Development Unit, Cropping Systems Research Laboratory, U.S. Department of Agriculture-Agricultural Research Service, Lubbock, TX, United States; ^3^ Plant Gene Expression Center, U.S. Department of Agriculture-Agricultural Research Service, Albany, CA, United States

**Keywords:** EMS mutagenesis, bulk segregant analysis, whole-genome resequencing, flowering time, gene expression, photoperiodic flowering, *Sorghum bicolor*

## Abstract

**Introduction:**

A fundamental developmental switch for plants is transition from vegetative to floral growth, which integrates external and internal signals. INDETERMINATE1 (Id1) family proteins are zinc finger transcription factors that activate flowering in grasses regardless of photoperiod. Mutations in maize *Id1* and rice *Id1* (*RID1*) cause very late flowering. RID1 promotes expression of the flowering activator genes *Early Heading Date1* (*Ehd1*) and *Heading date 1* (*Hd1*), a rice homolog of CONSTANS (CO).

**Methods and results:**

Mapping of two recessive late flowering mutants from a pedigreed sorghum EMS mutant library identified two distinct mutations in the *Sorghum bicolor Id1* (*SbId1*) homolog, mutant alleles named *sbid1-1* and *sbid1-2*. The weaker *sbid1-1* allele caused a 35 day delay in reaching boot stage in the field, but its effect was limited to 6 days under greenhouse conditions. The strong *sbid1-2* allele delayed boot stage by more than 60 days in the field and under greenhouse conditions. When *sbid1-1* and *sbid1-2* were combined, the delayed flowering phenotype remained and resembled that of *sbid1-2*, confirming late flowering was due to loss of *SbId1* function. Evaluation of major flowering time regulatory gene expression in *sbid1-2* showed that *SbId1* is needed for expression of floral activators, like *SbCO* and *SbCN8*, and repressors, like *SbPRR37* and *SbGhd7*.

**Discussion:**

These results demonstrate a conserved role for *SbId1* in promotion of flowering in sorghum, where it appears to be critical to allow expression of most major flowering regulatory genes.

## Introduction

Plant flowering behavior is determined by transcriptional and posttranscriptional signaling networks that promote flowering under inductive photoperiods and also repress flowering under non-inductive photoperiods. A conserved integration point for flowering signals is the *CONSTANS* (*CO*) - *FLOWERING LOCUS T* (*FT*) regulatory module (Song et al., 2015), named for genes first discovered in *Arabidopsis thaliana*. *CO* encodes a transcription factor with B-box domains and a signature CO, CO-LIKE and TIMING OF CAB1 (CCT) domain that is a member of a widely conserved family in plants (Putterill et al., 1995; Griffiths et al., 2003). Arabidopsis FT protein is part of the larger plant PEBP-related family conserved throughout flowering plants containing FT-like florigen-related proteins (Danilevskaya et al., 2008; Turck et al., 2008). Leaf expressed FT-like proteins in Arabidopsis, rice, tomato, and cucurbits serve as molecular florigens that stimulate floral development at the shoot apical meristem (Lifschitz et al., 2006; Jaeger and Wigge, 2007; Lin et al., 2007; Tamaki et al., 2007; Notaguchi et al., 2008). The primary role of CO is regulation of *FT* expression. Whether CO protein activates or represses its *FT* target genes varies among plants. In Arabidopsis, *CO* activates *FT* expression under floral inductive long-day (LD) photoperiods to promote flowering (Samach and Coupland, 2000; Valverde et al., 2004; Song et al., 2012). The rice *CO* ortholog *Heading date 1* (*Hd1*) upregulates expression of the *FT* ortholog *Heading date 3a* (*Hd3a*) in floral inductive SD photoperiods but is a repressor in LD (Yano et al., 2000; Kojima et al., 2002). Hd1 potentially adopts its repressive function by participating in a co-repressor complex with the LD-expressed flowering repressor rice *PSEUDORESPONSE REGULATOR 37* (Os*PRR37*) (Koo et al., 2013). In this role, *Hd1* also represses the expression of rice *Early heading date 1* (*Ehd1*). *Ehd1* encodes a B-type response regulator that promotes *Hd3a* expression under SD separate from *Hd1* (Doi et al., 2004; Itoh et al., 2010; Zhao et al., 2015). *CONSTANS OF Zea mays1* (*CONZ1*) is an ortholog of rice *Hd1* (Miller et al., 2008) proposed to regulate the maize florigen-related gene *Zea mays CENTRORADIALIS 8* (*ZCN8*). ZCN8 is a presumed florigen, because silencing *ZCN8* expression delays flowering in maize (Meng et al., 2011) and transgenic phloem-specific expression of *ZCN8* in Arabidopsis produces early flowering in an *ft* mutant background (Lazakis et al., 2011).

Sorghum *CONSTANS* (*SbCO*) acts upstream to promote the expression of *SbEhd1* and sorghum *CENTRORADIALIS 8* (*SbCN8*), the co-linear ortholog of maize *ZCN8*, and *SbCN12*, a PEBP-family gene orthologous between maize and sorghum (Murphy et al., 2011; Yang et al., 2014b). Both *SbCN8* and *SbCN12* possess florigen activity when overexpressed in Arabidopsis (Wolabu et al., 2016). Collectively, *SbCN8* and *SbCN12* are regulated by *SbCO* and *SbEhd1* (Murphy et al., 2014; Yang et al., 2014b), consistent with this set of genes acting as the CO-FT module in sorghum.

Additional upstream regulators of *SbCO* and *SbEhd1* are the repressors sorghum *PRR37* (*SbPRR37*) (Murphy et al., 2011) and sorghum *Grain number, plant height, and heading date* (*SbGhd7*) (Murphy et al., 2014). *SbPRR37* encodes a type of transcriptional repressor originally discovered as core circadian clock genes in Arabidopsis (Farre and Liu, 2013), but *SbPRR37* has no contribution to sorghum circadian clock function (Murphy et al., 2011). Differentially functional *SbPRR37* alleles underlie the flowering time-associated *Maturity* locus *Ma1*, which has the largest impact on sorghum flowering time (Quinby, 1945; Quinby, 1967). As is typical of PRR37 proteins, SbPRR37 contains a CCT domain like Hd1/CO, but it belongs to a distinct protein subfamily (Murakami et al., 2003; Murphy et al., 2011). SbGhd7 is a homolog of rice Ghd7 discovered as a quantitative trait locus contributing to natural variation in heading date (Xue et al., 2008). Ghd7 proteins also contain a CCT domain but do not contain the B-box domains found in Hd1/CO (Murphy et al., 2014). Genetic variation at *SbGhd7* corresponds to sorghum *Maturity* locus *Ma6* (Rooney and Aydin, 1999; Murphy et al., 2014). *SbPRR37* and *SbGhd7* inhibit flowering under LD conditions, by repressing the expression of flowering activators, primarily *SbEhd1* but also *SbCO*, to ultimately repress the expression of florigen-related genes like *SbFT*, *SbCN8*, and *SbCN12* (Murphy et al., 2011; Murphy et al., 2014). Under LD, SbPRR37 is also proposed to interact with SbCO as part of a repressor complex that turns SbCO into an inhibitor of florigen gene expression (Yang et al., 2014b).

Genes in the *INDETERMINATE1* (*Id1*) family act as activators of flowering in grasses. The maize *id1* mutant is very late flowering (Singleton, 1946; Colasanti et al., 1998). Mutations in the rice *Id1* homolog, *RID1*, cause a never-flowering phenotype (Wu et al., 2008). RID1 contributes to stimulation of *Ehd1* expression to promote flowering in LD and SD photoperiods (Matsubara et al., 2008; Park et al., 2008). Id1 proteins belong to a family of transcription factors characterized by a unique arrangement of four Cys2His2-type zinc finger motifs (Colasanti et al., 2006). Maize Id1 protein binds to DNA *in vitro* at an 11-bp DNA sequence motif (Kozaki et al., 2004). RID1 has been demonstrated to bind promoter regions of rice *Hd3a* and *RFT1 in vitro* (Deng et al., 2017). Also, ChIP-seq analysis with RID1 demonstrated it binds upstream of several flowering time genes, including rice *Hd1* (Zhang et al., 2022b). Comparison of chromatin modifications in the maize *id1* mutant to the wild type indicated that Id1 influences the chromatin state around florigen genes *ZCN8* and *ZCN7*, which is apparent as changes in histone acetylation (Mascheretti et al., 2015). Similarly, RID1 is involved in regulating chromatin accessibility and histone methylation at the promoters of *Hd3a* and *RTF1* (Zhang et al., 2022a).

To identify genes that promote flowering in sorghum, late-flowering mutants were identified in a pedigreed sorghum EMS mutant library in the BTx623 genetic background (Jiao et al., 2016). Two independent late-flowering mutants discovered in this population each substantially delayed the timing of boot stage, an early visual indicator of flowering. The causal mutation in each line was identified by whole-genome resequencing and with preexisting sequence knowledge of the EMS mutations present in the parental population. In each case, the causal mutation was in the sorghum *Id1* (*SbId1*) homolog. The *sbid1-1* allele is a non-synonymous mutation that changes a conserved amino acid in the second zinc finger domain of SbId1. The *sbid1-2* allele is a nonsense mutation that results in a predicted SbId1 protein approximately half its normal size. Plants carrying the weaker *sbid1-1* eventually flowered in the field, but plants with the *sbid1-2* allele did not. The flowering delay in *sbid1-1* was reduced when plants were grown under greenhouse conditions to nearly that of wild-type plants. When *sbid1-1* and *sbid1-2* were combined, the flowering phenotype was that of *sbid1-2.* Evaluation of major flowering time regulatory gene expression in *the sbid1-2* background showed that *Id1* is needed for expression of floral activators, like *SbCO* and *SbCN8*, and repressors, like *SbPRR37* and *SbGhd7*. These results demonstrate a conserved role for *SbId1* in promotion of flowering in sorghum, where it appears to be critical to allow expression of most major regulatory genes.

## Methods

### Plant lines and environmental conditions

All sorghum lines were the BTx623 genetic background carrying the *ms8* allele (Xin et al., 2017). The M2-1299 and M2-0483 lines were from a collection of 256 whole-genome-sequenced M4 EMS-mutagenized sorghum lines described by Jiao et al. (2016). Plants were screened for the *sbid1-1 and sbid1-2* mutations in *SbId1* by Derived Cleaved Amplified Polymorphic Sequences PCR with the primers in **Supplementary Table 3** together with restriction enzymes *Hpy188I* and *HpyCH4V*, respectively, from New England Biolabs (neb.com). The *Hyp188I* restriction enzyme cleaves the *sbid1-1* mutant-derived PCR fragment, but not the wild-type PCR fragment, and the *HpyCH4V* restriction enzyme does not cleave the *sbid1-2* mutant-derived PCR fragment but cleaves the wild-type PCR fragment.

Greenhouse flowering time trials were under LD conditions of 16-h days and 8-h nights. Natural sunlight was supplemented with LED lighting from either LumiGrow Pro325 (lumigrow.com) or DayBreak LED GrowLuXx (daybreakled.com) fixtures. Daytime temperature was set to 26°C, and nighttime temperature was set to 20°C. Seedlings were sown in 4-in. peat pots filled with SuperSoil from The Scotts Company (scotts.com) and transplanted to 13-L pots filled with corn soil (composed of aged wood fines, green waste compost, fir bark, grape compost, rice hulls, chicken manure, red lava, and sandy loam mixed by American Soil and Stone (Richmond, CA)) when seedlings reached the three-to-four-leaf stage (10 to 15 days old). Greenhouse plants were watered twice daily and received 20–20–20 N–P–K fertilizer once a week. These trials were conducted over the same season as field trials, beginning in late May and finishing in late September. The exceptions were experiments allowing *sbid1-2* or *sbid1-2*/*id1-1* lines to flower and set seed that extended into December and January.

Field-grown plants for flowering time trials were grown at the University of California, Davis Vegetable Crops facility in Davis, CA, during the summers of 2019–2023. Plants were started by seed sown directly to soil in rows that were watered and fertilized by drip irrigation. Field season began in late May and finished late September or early October.

Plants for analysis of leaf gene expression were grown to the four-leaf stage (approximately 2 weeks old) in peat pots under standard greenhouse conditions, and then pots were transferred to Percival Scientific model PGC-36C9 growth chambers (percival-scientific.com) set to a light:dark cycle of 16-h light: 8-h dark. The light period was 26°C, and the dark period was 22°C. Light was provided by white LEDs (wavelength range 400–700 nm) at a total fluence rate of 360 µM photons/m^2^ s. Plants were watered by subirrigation twice a week, once with water and once with a water solution containing 134 ppm of Peters Professional 20–20–20 fertilizer (ICL, icl-sf.com).

### BSA-seq mapping of *sbid1-1* and *sbid1-2* alleles

Genomic DNA was prepared from leaf tissue taken from late-flowering mutant plants with the Qiagen DNeasy Plant Mini kit according to the manufacturer’s recommendations (qiagen.com). The genomic DNA was precipitated with 1/10 volumes of 3 M sodium acetate and 2.5 volumes of 100% ethanol to remove contaminating salts, the DNA pellet dissolved in Ambion nuclease-free water (thermofisher.com), and the DNA concentration determined with the Qubit dsDNA Quantification Assay Kit (thermofisher.com).

A genomic DNA pool for deep sequencing was made by combining 500 ng of genomic DNA from 20 mutant individuals. Sequencing library construction and deep sequencing were performed by Novogene (novogene.com). Briefly, DNA was fragmented by sonication, poly-A tailed, ligated to adapters for Illumina sequencing, and PCR amplified with Illumina primers P5 and P7 index oligos. PCR fragments were purified with the AMPure XP system (beckman.com) and the library size distribution checked by Agilent 2100 Bioanalyzer (agilent.com) and quantified by qPCR. Libraries were 150-bp pair-end sequenced on an Illumina NovaSeq 6000 machine to greater than 10X genome coverage. The Illumina sequencing FASTQ files were deposited at the NCBI Sequence Read Archive (SRA) and are available at accession numbers SRR25730657 (https://www.ncbi.nlm.nih.gov/sra/SRR25730657) and SRR25668575 (https://www.ncbi.nlm.nih.gov/sra/SRR25668575) (Xin and Harmon, 2023). The BSA-seq workflow was run online on the cloud-based analysis platform SciApps (sciapps.org) with default settings according to the protocol described by Wang et al. (2021) using *Sorghum bicolor* genome assembly v3.0.1 and annotation v3.0 from Phytozome v13 (phytozome-next.jgi.doe.gov/).

### Analysis of flowering time gene expression by qPCR

Plants at the leaf 5 stage (approximately 21 days old) were sampled by cutting across the leaf 5 ligule with a razor blade. All the tissue extending above the ligule was collected, the midvein removed from mature leaves with a razor blade, wrapped in aluminum foil, flash frozen by immersion in liquid nitrogen, and stored at −80°C. Samples were taken at 4-h intervals from plants under a regular day–night cycle or an opposite day–night cycle, so that each sampling time collected two time points separated by 12 h. Samples at ZT0 were collected after lights in the growth chamber turned on and samples at ZT16 were collected after lights turned off. Two individual plants were collected per time point in two independent experiments, to generate a total of four biological replicates for each genotype. White LED headlamps covered with two layers of Roscolux 89 (Moss Green; us.rosco.com) filter were used to aid tissue collection under a green safelight.

Tissue samples were hand-ground under liquid nitrogen with a mortar and pestle. Total RNA was isolated from approximately 100 mg of tissue with the Qiagen RNeasy Plant Mini Kit and on-column DNase I digestion with the Qiagen RNase-Free DNase Set according to the manufacturer’s recommendations (qiagen.com). Total RNA was precipitated with 1/10 volumes of 3 M sodium acetate and 2.5 volumes of 100% ethanol to remove contaminating salts and the RNA pellet dissolved in Ambion nuclease-free water (thermofisher.com). RNA concentration was determined with a NanoDrop spectrophotometer (nanodrop.com). First-strand cDNA was synthesized from 2 µg of total RNA with Thermo Fisher Scientific Maxima Reverse Transcriptase and oligo(dT)_18_ according to the manufacturer’s recommendations (thermofisher.com). The final products were diluted with four volumes of Milli-Q water (EMD Millipore, Hayward, CA), and this served as a template for qPCR with the primers listed in **Supplementary Table 3**.

Two technical replicate qPCR reactions were composed and performed as described previously (Bendix et al., 2013). C_q_ values were calculated with the regression function for each primer set in Bio-Rad CFX Manager Software (Bio-Rad, Hercules, CA). Relative transcript level was calculated as 2^(C_q_^normalizer^ − C_q_^experimental^), where C_q_^normalizer^ is the geometric mean of the C_q_ values for the normalizer 1 and normalizer 2 primer sets (**Supplementary Table 3**). Relative expression level was relative transcript level normalized to the average relative transcript level for all timepoints, genotypes, and replicates.

### Identification of normalizer transcripts for qPCR

The normalizer transcripts for qPCR analysis were selected based on constant temporal and uniform leaf expression characteristics in public RNA-seq datasets. First, transcripts were identified that displayed moderate expression that was constant across the 24 samples in a 72-h diurnal time series made from leaves of the BTx623 inbred line (Lai et al., 2020). Transcripts with an average FPKM value between 25 and 500 were selected as a moderately expressed set. Transcripts from this moderately expressed set were selected for constant diurnal expression based on a JTK_CYCLE (Hughes et al., 2010) adjusted p-value of 1.0, a percent coefficient of variation <8, and a ratio of highest/lowest expression <1.4. The percent coefficient of variation was calculated as %CV = ((standard deviation/average FPKM) × 100). This constant diurnal expression set contained 27 genes. Second, the genes from the constant diurnal expressed set were filtered for uniform expression in RNA-seq libraries from 197 leaf samples from the BTx623 and RTx430 inbred lines made as part of the 2016 EPICON field trial (Varoquaux et al., 2019). Uniform expression was determined based on a percent coefficient of variation <30 and a ratio of highest/lowest expression <7 for normalized counts for all leaf samples. The primers in **Supplementary Table 3** targeting transcripts from genes *Sobic.008G153500* and *Sobic.010G068400* were found to exhibit 95%–105% efficiency and uniform Cq values with cDNA from a range of tissues and times of day.

## Results

### Identification of the *sbid1-1* allele, an EMS mutation with a conditional late-flowering phenotype

Screening of a pedigreed sorghum EMS mutant library (Jiao et al., 2016) for visual growth and developmental phenotypes identified a late-flowering individual from the 15M2-1299 line. This plant was crossed to the wild-type *male sterile 8 (ms8)* line (Xin et al., 2017) to generate material for mapping the causal mutation. The flowering behavior of the selfed mutant and the F1 *ms8*/mutant progeny was then evaluated in the field at Davis, California. Flowering was scored as the number of days from sowing to boot stage, an early visual indication of flowering. Plants were considered at boot stage when the collar of the flag leaf appeared in the whorl. The F1 progeny and wild-type control plants both flowered after an average of 51 (± 4.3 standard deviation, SD) and 52 (± 4.1 SD) days, respectively, whereas mutant progeny reached boot stage after an average of 95 (± 7.9 SD) days (**Supplementary Figure 1A**). Next season, the F2 progeny were grown out to confirm the late-flowering phenotype and to collect samples for mapping the position of the mutation. Out of 72 F2 plants, 20 individuals were late flowering, reaching boot stage more than 40 days later than their siblings (**Supplementary Figure 1B**). The 20 late-flowering individuals from this trial were used as the mapping population to identify the EMS mutation responsible for the later-flowering phenotype.

Genome resequencing and bulk segregant analysis (BSA) mapping associated the late-flowering phenotype with an EMS-derived lesion in a sorghum *Id1* (*SbId1*) homolog. A genomic DNA pool from the 20 F2 late-flowering individuals was deep sequenced, and these data were used in the BSA-seq mapping pipeline (Wang et al., 2021). This approach takes advantage of the existing database of positional information and mutation effect predictions for EMS-derived small nucleotide polymorphisms (SNPs) in the mutant library to identify SNPs linked with the mutant phenotype and determine candidate non-synonymous, deleterious mutations. A 45.5-Mb region on chromosome 1 had SNPs linked to the flowering phenotype (**Supplementary Figure 2A**). Within this region were 10 EMS-derived non-synonymous mutations within coding regions of genes and above a linking probability of 5. Five of these mutations were predicted to be deleterious to gene function (**Supplementary Table 1**). Of these potentially deleterious mutations, an obvious candidate was the C to T transition within *SbId1* (*Sobic.001G242900*) that resulted in the substitution of a conserved proline at position 155 with a serine (P155S) within the second zinc finger domain (**Supplementary Figures 2**, **3**, and **Supplementary Table 1**). This potential *SbId1* mutant allele was named *sbid1-1*.

**Figure 1:**
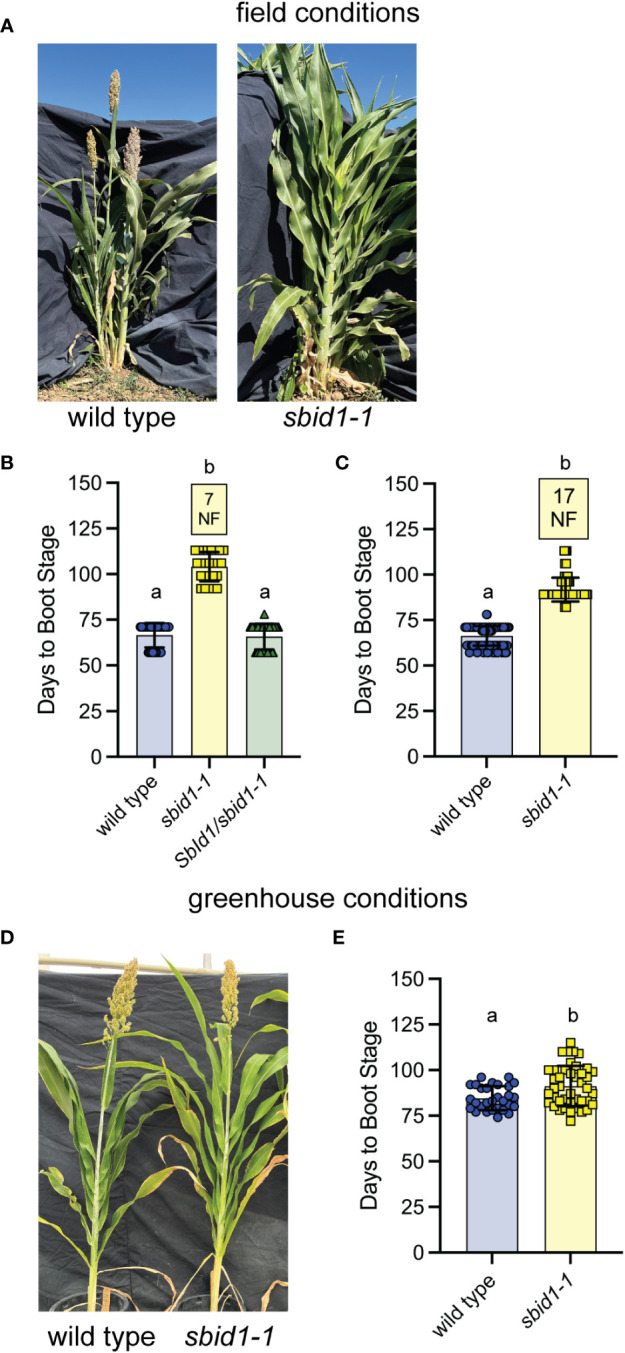
Homozygous *sbid1-1* plants have a more pronounced flowering time delay under field conditions. **(A)** Representative wild-type (BTx623 inbred) and *sbid1-1* plants at 109 days from sowing in the field. **(B, C)** Days to boot stage from sowing in field conditions at Davis, CA. Data are combined from two field trials. **(B)** Wild type (n = 28), *sbid1-1* (n = 25), and *SbId1*/*sbid1-1* heterozygotes (n = 60) from selfed *sbid1-1* heterozygous parent. **(C)** Wild-type (n = 150) and *sbid1-1* plants (n = 166). “NF” in yellow-colored boxes indicates the number of plants that had not flowered in at least 113 days after sowing. **(D)** Representative wild-type (BTx623 inbred) and *sbid1-1* plants grown under greenhouse conditions at 107 days from sowing. **(E)** Days to boot stage from sowing for wild-type (n = 29) and *sbid1-1* (n = 42) plants under greenhouse conditions. Means sharing a common letter are not significantly different by unpaired two-tailed t-test at the p <0.05 level of significance. Error bars are ± standard deviation.

In subsequent field trials, the *sbid1-1* mutation cosegregated in backcross 1 (BC1) F2 populations with a late-flowering phenotype like that of the original mutant line. Consistent with a recessive allele, plants homozygous for *sbid1-1* reached boot stage at an average of 104 (± 8.0 SD) days, which was later than plants genotyping as heterozygous for this mutation and wild type that reached boot stage at an average of 66 days (± 7.0 SD) (**Figures 1A, B**). In the same field trials, two different plantings of a homozygous mutant BC1F3 line required an average of 92 (± 6.6 SD) days to begin booting when all wild-type plants achieved boot stage within an average of 66 (± 5.4 SD) days (**Figure 1C**). Also, 17 out of 123 plants did not boot upon termination of the trial at 113 days after sowing. These results indicate that the mutation corresponding to the *sbid1-1* allele is associated with a significant delay in flowering.

Flowering time trials in the greenhouse revealed that the effect of the *sbid1-1* allele is weaker under these conditions. Across three independent greenhouse trials starting in May to June of 2021–2023, *sbid1-1* reached boot stage at an average of 91 (± 11 SD) days, which was 6 days later than the wild-type average of 85 (± 6.5 SD) days (**Figures 1D, E**), instead of the >30-day delay apparent in the field trials. These results show that the flowering delay caused by *sbid1-1* flowering was greatest in the field, and this effect was almost fully mitigated by unknown aspects of growth under greenhouse conditions.

### Identification of the EMS-derived *sbid1-2* allele that essentially blocks flowering

Plants carrying the late-flowering mutation in the 15M2-0483 EMS line were discovered in an F2 population from a backcross of an unrelated mutant (with normal flowering behavior) to the *ms8* BTx623 line. Tissue samples were taken from 20 late-flowering individuals in this population to capture their genetic information, since the extreme late-flowering phenotype of these plants could have potentially interfered with recovery of seeds. Genomic DNA was prepared from these plants for mapping the position of the causal mutation with the BSA-seq pipeline. Mapping based on sequence information from this genomic DNA pool identified SNPs from regions on chromosomes 1, 4, 7, and 10 as potentially linked to the late-flowering phenotype (**Supplementary Figure 2B**). BSA-seq called three non-synonymous mutations as linked at a linking probability of 5 with the only one predicted to be deleterious in a gene of unknown function (**Supplementary Table 2**). However, just under the significance threshold was a C to T transition in *SbId1* that produced a nonsense mutation replacing the glutamine at position 199 with a stop codon (Q199*) in the third zinc finger domain (**Supplementary Figure 2B**). This mutation truncates the 428 amino acid ID1 protein to approximately half its usual length (**Supplementary Figure 3**). This putative Sb*Id1* mutant allele was named *sbid1-2*.

**Figure 2:**
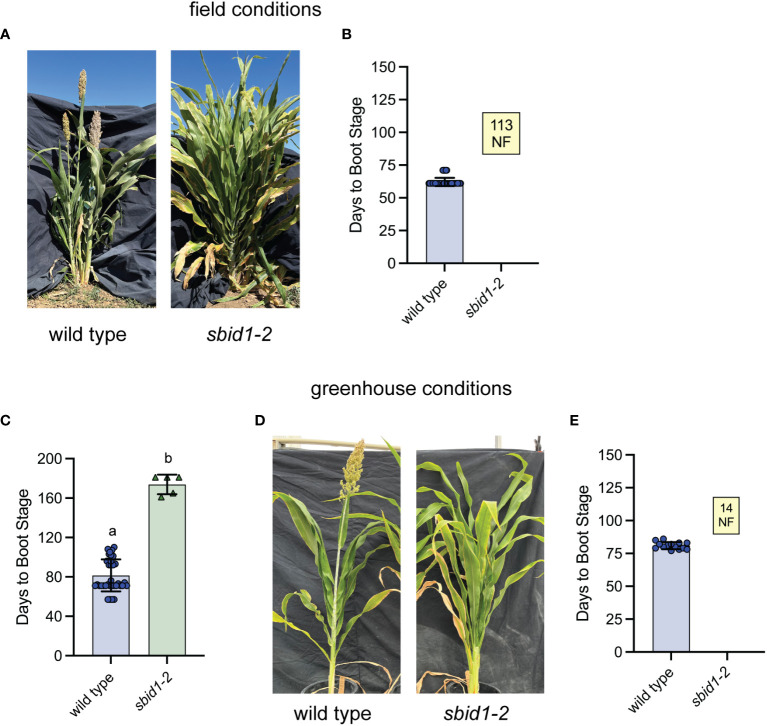
Homozygous *sbid1-2* plants do not achieve boot stage under standard field or greenhouse growth conditions. **(A)** Representative wild-type (BTx623 inbred) and *sbid1-2* plants at 109 days from sowing in the field. **(B)** Days to boot stage from sowing for wild type (n = 142) and *sbid1-2* (n = 113) in separate two field trials in Davis, CA. **(C, E)** Days to boot stage from sowing under greenhouse conditions. **(C)** Wild type (n = 36) and *sbid1-2* (n = 5) in a trial where *sbid1-1* plants were allowed to reach boot stage and set seed. **(D)** Representative wild-type (BTx623 inbred) and *sbid1-2* plants grown under greenhouse conditions at 107 days from sowing. **(E)** Wild-type (n = 14) and *sbid1-2* (n = 14) plants. “NF” in the yellow-colored box indicates the number of plants that had not flowered in at least 120 days after sowing. Means sharing a common letter are not significantly different by unpaired two-tailed t-test at p < 0.05 level of significance. Error bars are ± standard deviation.

To confirm that the *sbid1-2* mutation was linked to the flowering phenotype, 15M2-0483 F2 populations were grown out in the field and greenhouse, and plants were screened by PCR for the *sbid1-2* mutation and were scored for days to reach boot stage. Out of 39 plants in the field, the four plants genotyping as homozygous for *sbid1-2* did not boot after 140 days, whereas all plants genotyping as heterozygous or wild type at this position in *Id1* reached boot stage within 71 days. This result indicates that *sbid1-2* is a recessive allele. In two subsequent field trials that began 17 days apart in Davis, CA, none of the 113 *sbid1-2* mutant plants reached boot stage after 113 days from sowing, but all 142 wild-type plants reached this stage with a population average of 62 (± 3.2 SD) days (**Figures 2A, B**).

The flowering delay associated with the *sbid1-2* allele was also profound under greenhouse conditions. In the first greenhouse trial, the four *sbid1-2* homozygous plants recovered did not boot after terminating the experiment at 150 days, whereas the average days to boot stage for eight heterozygous plants was 90 (± 4 SD) days, only 7 days later than the wild-type average of 83 (± 7 SD) days. In the next greenhouse trial, five homozygous *sbid1-2* plants were maintained as long as was required to reach boot stage and set seed. The average booting time for these mutant plants was 173 (± 10 SD) days with the earliest being 161 days and the latest 181 days (**Figure 2C**). The average boot time for the wild type in this trial was 81 (± 16 SD) days. The progeny of these homozygous *sbid1-2* mutant plants in the greenhouse did not achieve boot stage after more than 110 days, whereas the wild type required an average of 81 (± 2.7 SD) days (**Figures 2D, E**). These results demonstrate that plants homozygous for this C to T transition in *SbId1* have a profound flowering delay representing effectively a block in flowering for the length of typical field trials, but *sbid1-2* plants eventually flower when maintained for approximately 170 days in the greenhouse.

### Combining the *sbid1-1* and *sbid1-2* allele demonstrates non-complementation, confirming flowering requires *SbId1* activity

Complementation tests were performed to confirm that the *sbid1-1* and *sbid1-2* mutations were responsible for delaying flowering in each mutant background. F1 plants with the *sbid1-1* and *sbid1-2* alleles combined were generated by pollinating a male sterile *sbid1-2* heterozygote with pollen from an *sbid1-1* homozygous plant. F2 populations were made by allowing F1 plants to self in the greenhouse.

**Figure 3:**
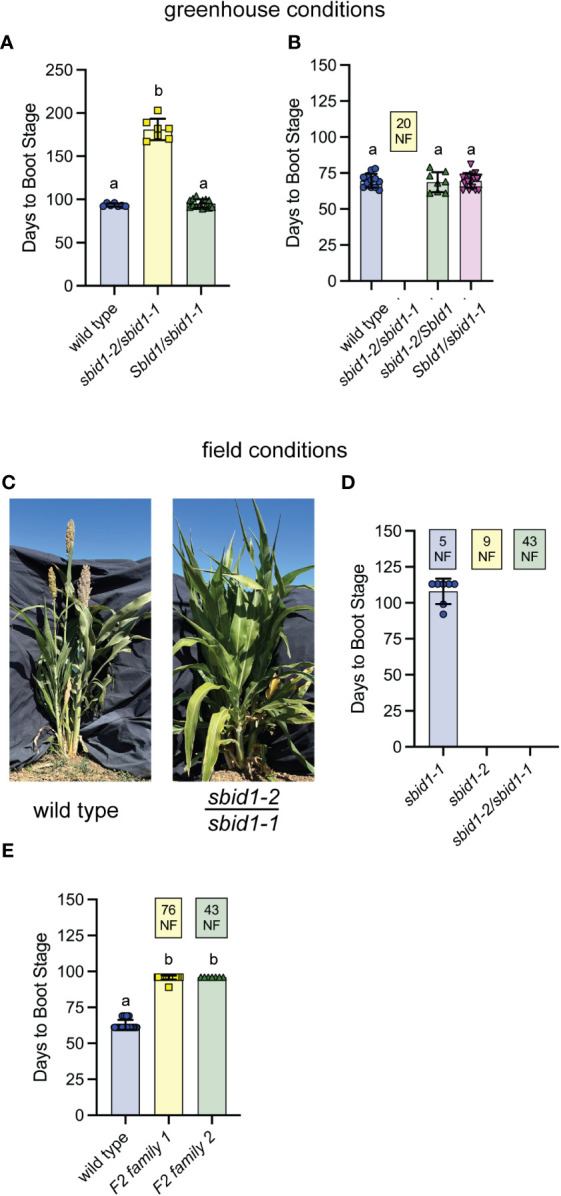
Non-complementation by *sbid1-1* and *sbid1-2* alleles confirms *SbId1* is needed to promote flowering. **(A, B)** Days to boot stage under greenhouse conditions in 2022 **(A)** and 2023 **(B)**. **(A)** Wild-type (BTx623) (n = 7), *sbid1-2*/*sbid1-1* (n = 7), and *SbId1/sbid1-1* (n = 14) from pollination of a male sterile (*ms8*) *sbid1-2* heterozygous panicle with *sbid1-1* homozygous pollen. All plants were allowed to reach boot stage. **(B)** Wild-type and *sbid1-2*/*sbid1-1* (n = 20), sbid1-2/SbId1 (n = 8), and *SbId1*/*sbid1-1* (n = 18) from the pollination of a male sterile (*ms8*) *sbid1-2* heterozygous panicle with *sbid1-1* heterozygous pollen. “NF” in the yellow-colored box indicates the number of plants that had not flowered in at least 120 days after sowing. **(C)** Representative wild-type (BTx623 inbred) and *sbid1-2*/*sbid1-1* heterozygous plants at 109 days from sowing in the field. **(D, E)** Days to boot stage in the field at Davis, CA. **(D)** *sbid1-2*/*sbid1-1* (n = 43), *sbid1-1* (n = 12), and *sbid1-2* (n = 9) from selfed *sbid1-2*/*sbid1-1* plants. “NF” in colored boxes indicates the number of plants for that genotype that had not flowered in at least 113 days after sowing. The genotype of all plants in **(A, D)** was determined with PCR. **(E)** Wild type (n = 67) and individuals from either a pool of selfed *sbid1-2*/*sbid1-1* plants (F2 family 1; n = 100) and one selfed *sbid1-2*/*sbid1-1* plant (F2 family 2; n = 50). These plants were not tested for genotype at *SbId1*. “NF” in the colored box indicates the number of plants that had not flowered in at least 96 days after sowing. Means sharing a common letter are not significantly different by unpaired two-tailed t-test at p <0.05 level of significance. Error bars are ± standard deviation.

In the first greenhouse trial, F1 plants genotyping as *sbid1-2*/*sbid1-1* took an average of 181 (± 12.3 SD) days to reach boot stage, with a range of 167 to 203 days (**Figure 3A**). In contrast, F1 progeny genotyping as *SbId1/sbid1-1* reached boot stage at an average of 95 (± 4.9 SD) days, comparable with the average of 93 (± 12.7 SD) days for wild-type plants. The *sbid1-2*/*sbid1-1* behavior was like homozygous *sbid1-2* plants started at the same time and grown under the same conditions (**Figures 2C, E**). *sbid1-2*/*sbid1-1* F2 plants in a second greenhouse trial behaved similarly. The group of plants genotyping as either *SbId1/id1-1*, *sbid1-2/SbId1*, and wild type all reached boot stage at approximately 68 days, but none of the *sbid1-2/sbid1-1* plants did so after 100 days when the experiment was terminated (**Figure 3B**).

Comparable results were also obtained in field trials with PCR genotyped F2 plants. Out of a total of 64 progeny from two different self-fertilized *sbid1-1/sbid1-2* F1 plants, 7 of the 12 *sbid1-1/sbid1-1* flowered within the 113 days of the trial, but none of the 9 *sbid1-2/sbid1-2* or the 43 *sbid1-2*/*sbid1-1* plants had flowered (**Figures 3C, D**). Two additional F2 families from two different selfed *sbid1-2/sbid1-1* parents were grown out in the same field, but these plants were not genotyped at the *SbId1* locus. One family of 100 had 24 plants achieve boot stage after 96 days, whereas the second family of 50 had seven plants at boot stage after 96 days (**Figure 3E**). A wild-type population of 89 plants sown at the same time reached boot stage at an average of 63 (± 3.4 SD) days (**Figure 3E**). These results show that the mutations present in *SbId1* in the *sbid1-1* and *sbid1-2* alleles were responsible for delayed flowering. Furthermore, the severity of the *sbid1-2*/*sbid1-1* phenotype more closely matched the strong *sbid1-2* allele than the weaker *sbid1-1* allele, which was most evident under greenhouse conditions.

### The *sbid1-2* mutant allele significantly reduces expression of flowering time genes

**Figure 4:**
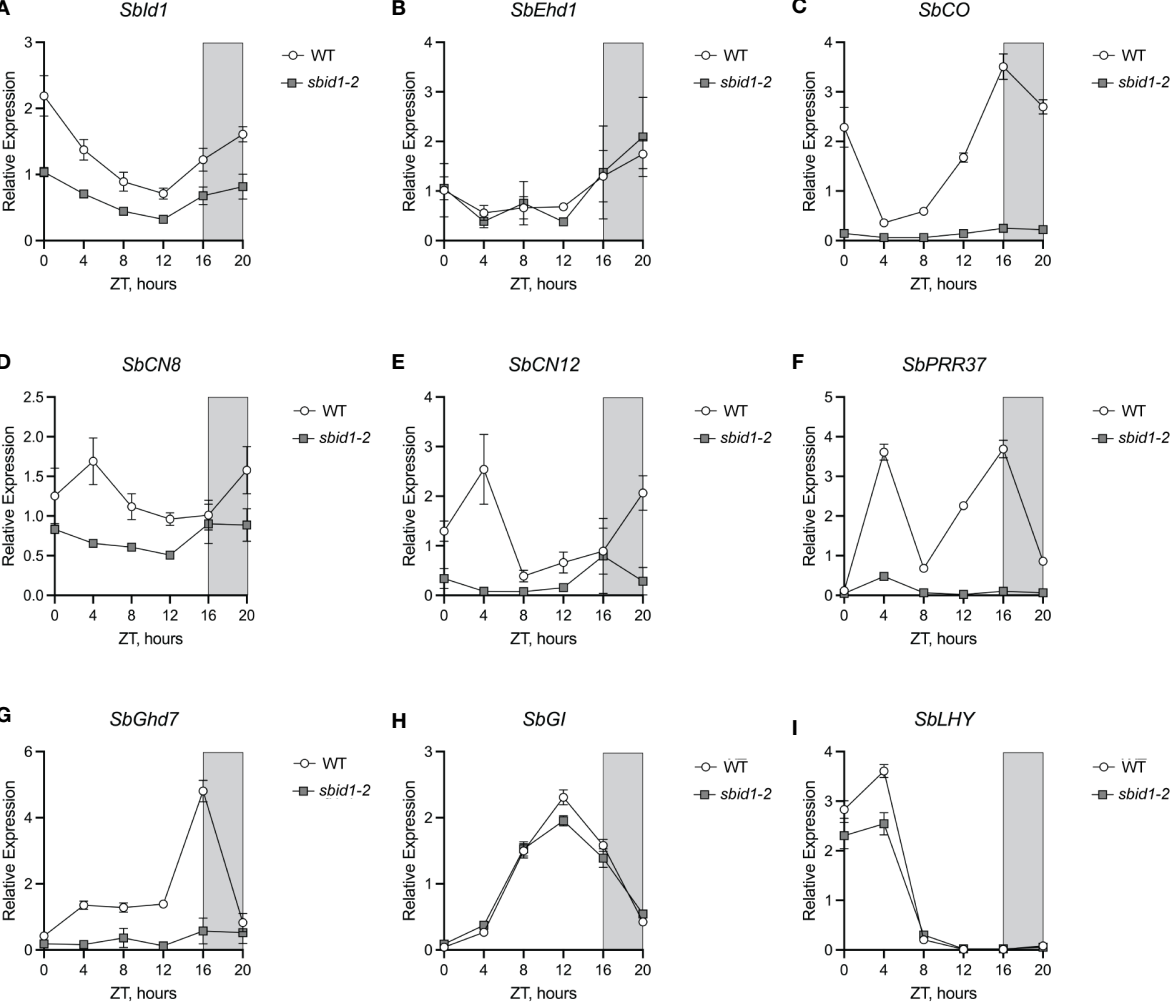
Loss of *SbId1* activity interferes with expression of major flower time activators and repressors. Relative expression of **(A)** *SbId1*, **(B)** *SbEhd1*, **(C)** *SbCO*, **(D)** *SbCN8*, **(E)** *SbCN12*, **(F)** *SbPRR37*, **(G)** *SbGhd7*, **(H)** *SbGI*, and **(I)** *SbLHY* in wild-type (white circles) or *sbid1-2* (gray squares) plants under LD conditions (16-h light and 8-h darkness). Zeitgeber time (ZT) in hours and ZT0 corresponded to lights on at dawn and ZT16 corresponded to lights off at night (gray shading). Points are the mean of four individual plants from two independent experiments. Error bars are ± standard error of the mean.

To gain insight into the origin of late flowering caused by loss of *SbId1* function, the effect of the *sbid1-2* allele on the expression level and daily accumulation pattern, or waveform, of major flowering time regulatory genes was examined in the leaves of plants at the leaf 5 stage (approximately 21 days old), at 4-h intervals over 24 h under LD conditions (i.e., 16-h days and 8-h nights). Evaluation of *SbId1* transcript levels in wild type and *sbid1-2* showed that levels were lower in the mutant at each timepoint, but the waveform was similar between mutant and wild type (**Figure 4A**). Since rice RID1 contributes to upregulation of the floral activator *Ehd1* under LD photoperiods (Matsubara et al., 2008; Park et al., 2008), levels of the *SbEhd1* transcript were determined for these time courses. No difference in *SbEhd1* levels was apparent between *sbid1-2* and wild type at any of the time points (**Figure 4B**). Also, the waveform for *SbEhd1* accumulation in *id1-2* matched the wild type, which was comparable with the *SbEhd1* waveform described previously for LD conditions (Murphy et al., 2011). In contrast, floral activator *SbCO* transcript accumulation in *sbid1-2* was low at all time points and lacked the wild-type waveform (Murphy et al., 2011) that rose beginning at 8 h after dawn (zeitgeber time (ZT) 8), peaked at the beginning of the dark period at ZT16, and extended to 4 h after dawn at ZT4 (**Figure 4C**). Expression of florigen-encoding genes *SbCN8* and *SbCN12* was also lower in *sbid1-2* than in the wild type, with a notable lack of peak expression at ZT4 (**Figures 4D, E**). These findings were consistent with a diminishment of *SbCN8* and *SbCN12* upregulation by *SbCO* in *sbid1-2* and, consequently, a block in promotion of the transition to flowering by these florigen genes.

Although the BTx623 genetic background has the inactive *sbprr37-3* (*ma1*) and *sbghd7-1* (*ma6*) alleles (Murphy et al., 2011; Murphy et al., 2014), the expression of *SbPRR37* and *SbGhd7* was compared between *sbid1-2* and the wild type to determine if *SbId1* contributes to transcriptional regulation of these major floral repressors. Both *SbPRR37* and *SbGhd7* transcripts were low at all time points in *sbid1-2* (**Figures 4F, G**). *SbPRR37* expression in *sbid1-2* lacked the major peaks at ZT4 in the morning and at ZT16 at the beginning of night apparent in the wild type (**Figure 4F**) and described previously for *SbPRR37* expression under LD (Murphy et al., 2011). Similarly, the primary *SbGhd7* peak at ZT16 in the wild type (Murphy et al., 2014) did not occur in *sbid1-2* (**Figure 4G**). These results show that *SbId1* contributes to activation of these floral repressor genes. The alteration in gene expression in *sbid1-2* was not due to a change in overall rhythmic gene expression, since expression waveforms for *SbGI*, a flowering regulator and circadian clock gene (Abdul-Awal et al., 2020), and *SbLHY*, a presumed circadian clock gene (Lai et al., 2020), were similar between sb*id1-2* and the wild type, except for reduced peak levels for both transcripts (**Figures 4H, I**). Thus, *Id1* is needed for expression of the major regulatory genes for flowering, including floral activators and repressors.

## Discussion

Two mutants with significant delays in flowering time discovered in a pedigreed sorghum EMS mutant library were identified as novel *sbid1* mutant alleles. Plants with the weaker *sbid1-1* non-synonymous mutation had booting delayed by more than 30 days in the field but behaved similar to the wild type under greenhouse conditions. The condition-sensitive nature of *sbid1-1* makes it a potentially useful tool to dissect the activity of *sbid1* in the future. The strong *sbid1-2* allele, a nonsense mutation that encodes for a truncated SbId1 protein, required approximately 170 days to reach boot stage, effectively blocking flowering under typical field conditions. Plants carrying *sbid1-1* and *sbid1-2* together had the strong flowering phenotype of *sbid1-2*, confirming that the phenotype arises from loss of *SbId1* function. Evaluation of expression for major flowering time regulatory genes in the *sbid1-2* background showed that *SbId1* is needed for expression of floral activators, like *SbCO* and its target genes *SbCN8* and *SbCN12*. Flowering time repressors *SbPRR37* and *SbGhd7* also had significantly reduced expression in the mutant background.

The strong flowering time delay caused by *sbid1-1* and *sbid1-2* mirrors the effect of *id1* and *rid1* mutants on flowering in maize (Singleton, 1946; Colasanti et al., 1998) and rice (Matsubara et al., 2008; Wu et al., 2008), respectively. However, analysis of the gene expression profile in *sbid1-2* highlights potential differences in or previously unknown aspects of the contribution of *SbId1* to the flowering time regulatory system. There was no indication that *SbId1* influences *SbEhd1* expression, unlike the case of *RID1* in rice (Matsubara et al., 2008; Park et al., 2008; Wu et al., 2008). Loss of *RID1* activity eliminates a peak in *Ehd1* expression that occurs at midday, which reduces Ehd1-promoted florigen gene expression (Matsubara et al., 2008; Park et al., 2008; Wu et al., 2008). Any impact of *sbid1-2* on *SbEhd1* expression may have been obscured by the lack of midday *SbEhd1* upregulation in the wild-type plants evaluated here. Regardless, a lack of *SbCO*, *SbCN8*, and *SbCN12* upregulation in *sbid1-2* indicates SbId1 protein promotes flowering through the SbCO-SbCN8/SbCN12 module. In this role, the SbId1 protein appears to be required to upregulate *SbCO* expression under LD conditions to promote *SbCN8* and *SbCN12* expression.

The fact that these *sbid1* alleles caused substantial flowering time delays in a genetic background lacking the activity of *SbPRR37* and *SbGhd7* demonstrates that *Id1* activity does not depend on these major flowering repressors. The BTx623 inbred carries the *sbprr37-3* allele of *ma1*, which encodes a Lys162Asp substitution in *SbPRR37* (Murphy et al., 2011), and the *sbghd7-1* allele of *ma6*, which is a 5-bp insertion in the *SbGhd7* coding sequence (Murphy et al., 2014). SbPRR37 and SbGhd7 delay flowering under LD conditions by repressing the expression of *SbEhd1* and inhibiting *SbCO* activation of florigen genes (Murphy et al., 2011; Murphy et al., 2014; Yang et al., 2014b).

Nevertheless, gene expression analysis indicated that *SbId1* is needed for expression of these repressor genes. *SbPRR37* and *SbGhd7* transcript levels were below basal levels in *sbid1-1*, with expression patterns lacking the peaks at midday (ZT8) and the transition from day to night (ZT16). Light signals mediated by phytochromes A and C, which represent maturity loci *Ma3* (Childs et al., 1997) and *Ma5* (Yang et al., 2014a), promote expression of *SbPRR37*and *SbGhd7* (Yang et al., 2014a). Therefore, it is conceivable that SbId1 plays a role in light-promoted upregulation of *SbPRR37* and *SbGhd7*. Similarly, rice *Ghd7* may be upregulated by RID1 (Wu et al., 2008), indicating that Id1 regulation of these major repressors is likely highly conserved.

These results demonstrate that *SbId1* potentially contributes to gene expression in all the major arms of flowering time regulation in sorghum to promote (Wu et al., 2008) upregulation of both activators like *SbCO* and *SbCN8*/*SbCN12*, as well as repressors *SbPRR37* and *SbGhd7*. Since RID1 and maize Id1 impact histone modifications to increase chromatin accessibility at the promoters of their respective florigen genes (Mascheretti et al., 2015; Zhang et al., 2022a), it is tempting to speculate that a general mechanism of Id1 protein action is establishment of active chromatin states at promoters of major regulatory genes to activate the photoperiod regulatory network. This model suggests that a conserved role for Id1 proteins is to establish the capacity for flowering time regulation, serving as a master regulator as proposed for RID1 in rice (Wu et al., 2008). In this role, SbId1 is needed for expression of both activators and repressors, not solely as an activator of downstream activators. Important tests of this idea are to evaluate the role of *SbId1* in genetic backgrounds like bioenergy sorghum accessions with intact systems for photoperiodic flowering time control, and to identify what changes, if any, mutations in *SbId1*, such as the alleles described here, have on the epigenetic states across the sorghum genome, most importantly at flowering time genes.

## Data availability statement

The datasets presented in this study can be found in online repositories. The names of the repository/repositories and accession number(s) can be found below: NCBI BioProject, PRJNA1006376, https://www.ncbi.nlm.nih.gov/bioproject/PRJNA1006376 NCBI SRA, SRR25730657, https://www.ncbi.nlm.nih.gov/sra/SRR25730657 NCBI SRA, SRR25668575, https://www.ncbi.nlm.nih.gov/sra/SRR25668575.

## Author contributions

SD: Formal analysis, Investigation, Writing – review & editing. JC: Investigation, Writing – review & editing. ZX: Investigation, Writing – review & editing, Formal analysis. FH: Conceptualization, Formal analysis, Funding acquisition, Investigation, Methodology, Supervision, Writing – original draft, Writing – review & editing.

## Supplementary material

Supplementary Figure 1 The mutation from the M2-1299 line significantly increases the number of days to reach boot stage.

Supplementary Figure 2 BSAseq results for mapping of late flowering alleles from M2-1299 and M2-0483.

Supplementary Figure 3 Alignment of Id1 homologs from C4 and C3 grasses.
