# Supplementary material for: Sorghum bicolor INDETERMINATE1 is a conserved primary regulator of flowering
Source: Front Plant Sci. 2023 Dec 13;14:1304822. doi: 10.3389/fpls.2023.1304822 (PMC10751353; doi:10.3389/fpls.2023.1304822)
Supplement: Supplementary Figure 1 — The mutation from the M2-1299 line significantly increases the number of days to reach boot stage. [file Image_1.pdf]

SUPPLEMENTAL FIGURES

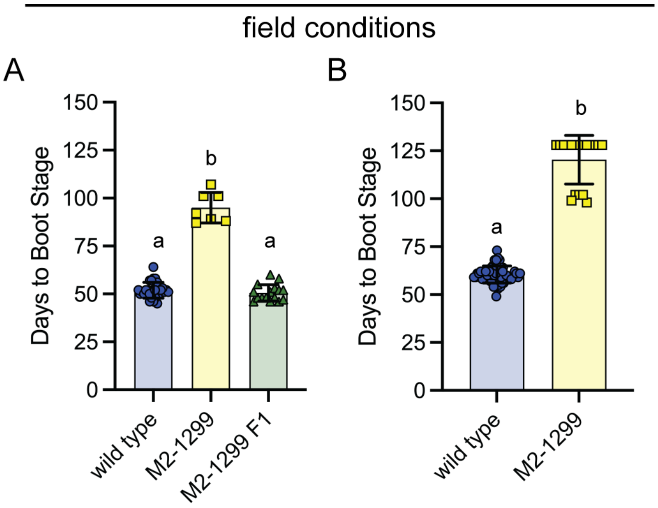

SFigure 1. The mutation from the M2-1299 line significantly increases the number of days to reach boot stage. A) Days to boot stage in the Davis, CA field for wild type (n = 28), M2_1299 late flowering line (n = 7), and F1 progeny from pollination of a male sterile (*ms8*) panicle with pollen from an M2-1299 late flowering individual (n = 16). B) Days to boot stage for wild type (n = 52) and 20 late flowering individuals out of 72 F2 progeny from the F1plants in (A). Means sharing a common letter are not significantly different by unpaired two-tailed t-test at $p < 0.05$ level of significance. Error bars are ± standard deviation.

A

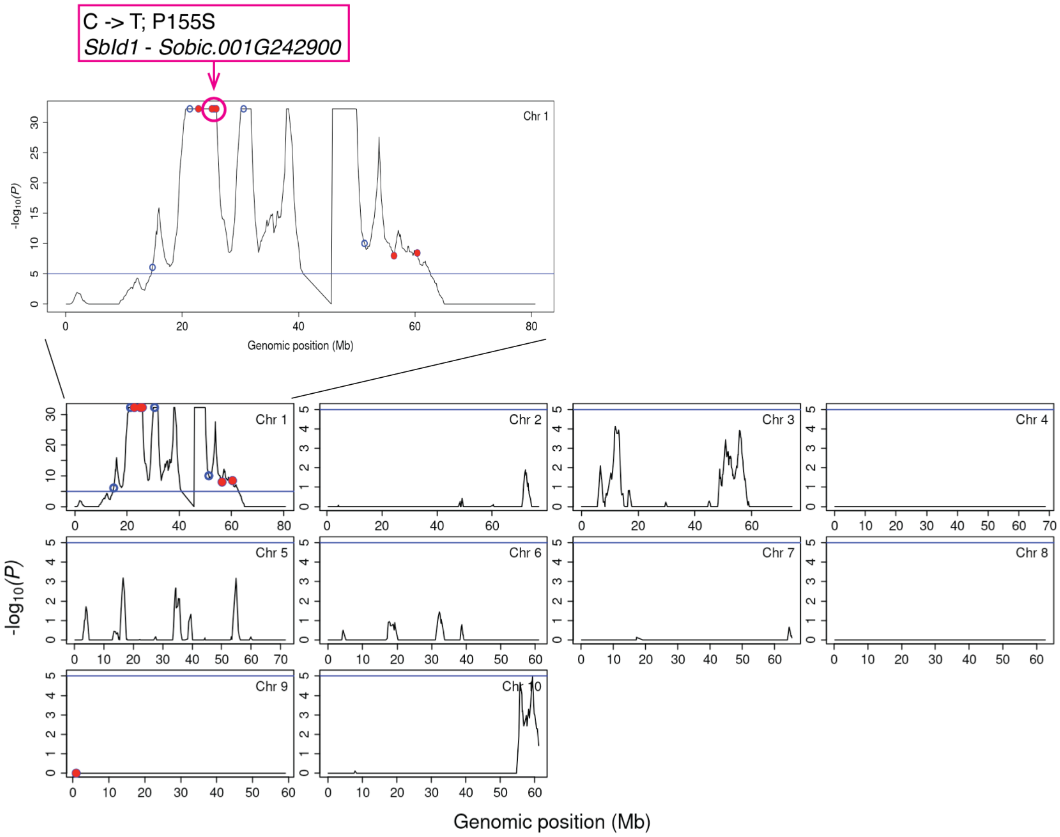
C -> T; P155S
SbId1 - Sobic.001G242900
Chr 1
-log10(P)
Genomic position (Mb)
Chr 1
Chr 2
Chr 3
Chr 4
Chr 5
Chr 6
Chr 7
Chr 8
Chr 9
Chr 10
-log10(P)
Genomic position (Mb)

B

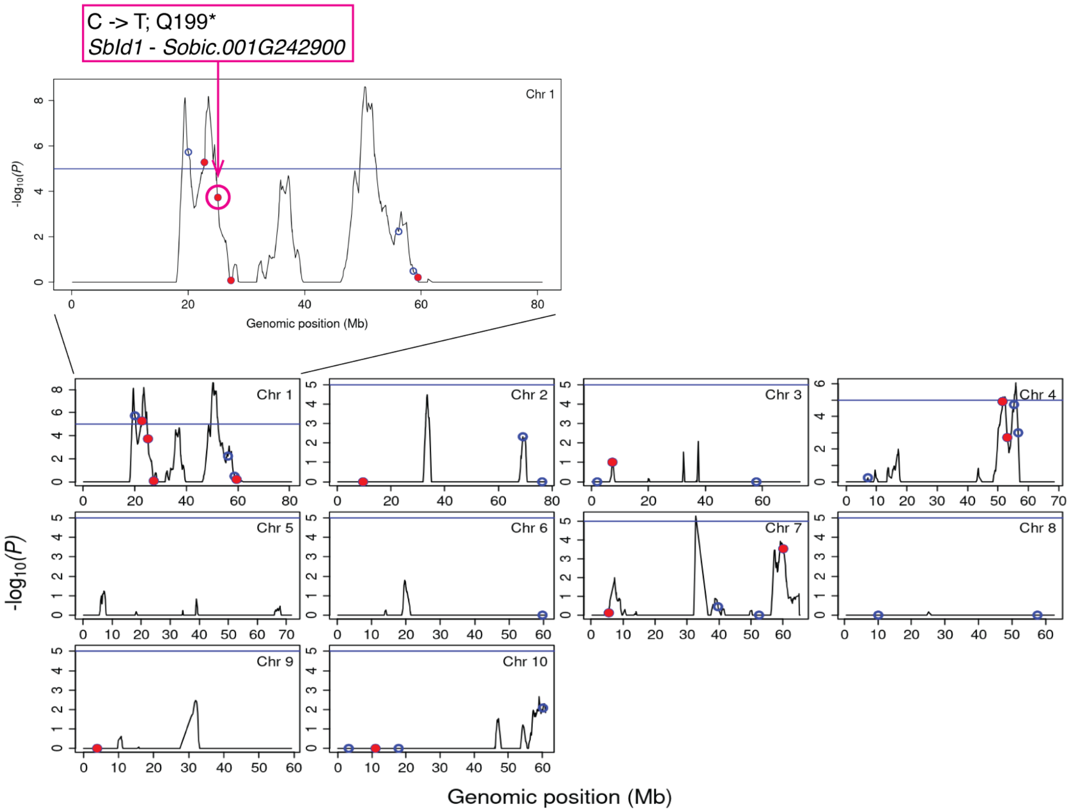
C -> T; Q199*
SbId1 - Sobic.001G242900
Chr 1
-log10(P)
Genomic position (Mb)
Chr 1
Chr 2
Chr 3
Chr 4
Chr 5
Chr 6
Chr 7
Chr 8
Chr 9
Chr 10
-log10(P)
Genomic position (Mb)

SFigure 2. BSAseq results for mapping of late flowering alleles from M2-1299 and M2-0483. Mapping of late flowering *sbid1-1* (A) and *sbid1-2* (B) allele in the *SbId1* gene by BSAseq method (1). BSAseq calculated linking probability P-value (-log10(P)) within a 5 megabase window across each of the 10 sorghum chromosomes. Blue circles indicate non-synonymous EMS-derived SNPs and red circles indicate mutations calculated by BSAseq to be significant. Blue horizontal line is P-value significance threshold $-\log_{10}(10^{-5})$. Plots above zoom in on details for chromosome 1. Magenta circle indicates the mutation in *SbId1* mutation and the box above lists details of the mutation and impact on SbId1 protein sequence.

```
Sobic.001G M-MMMLSDLSSEHEHEATGSSSYGG------DMASYALSPLFLAPAASATTVLPPAPP-H
GRMZM2G011 MQMMMLSDLSSD-DHEATGSSSYGG------DMASYALSPLFLAPAASATAPLPPPP---
Seita.9G24 ---MMLSDLSS--DHEATGSSSHGG------DIASYALSPLFLAPA-------PPLAG-A
Sevir.9G24 ---MMLSDLSS--DHEATGSSSHGG------DIASYALSPLFLAPA-------PPLAG-A
Bradi3g269 ---MMLSDLSS--DQEATGSNSHGGGGDVVLSNQHVLLNPLFAPAQHQQALLLRPAPQLE
LOC_Os10g2 M-------------------------------------------------------AA-A
                                                                   .

Sobic.001G QQPAEEEPNK--AAGTKRKRSQPGNPDPGAEVIALSPRTLVATNRFVCEICNKGFQRDQN
GRMZM2G011 QPPAEELTNKQAAGGGKRKRSQPGNPDPGAEVIALSPRTLVATNRFVCEICNKGFQRDQN
Seita.9G24 AAPQTEAP----RVGAKRKRSQPGNPDPGAEVIALSPRTLVATNRFVCEICNKGFQRDQN
Sevir.9G24 AAPQTEAP----RVGAKRKRSQPGNPDPGAEVIALSPRTLVATNRFVCEICNKGFQRDQN
Bradi3g269 QQP---------MAGGKRKRSQPGNPDPGAEVIALSPRALVATNRFVCEICNKGFQRDQN
LOC_Os10g2 QEPR-----------AKKKRSLPGNPDPEAEVIALSPRALVATNRFVCEVCNKGFQRDQN
             *             *:*** ****** *********:**********:**********
                                   sbid1-1 - 15M2-1299 - C->T - P155S
Sobic.001G LQLHRRGHNLPWKLRHRSSL--PSGSSGA--------RQQGGEAAAPRKRVYVCPEPTCV
GRMZM2G011 LQLHRRGHNLPWKLRQRSSLVVPSSSAAAGSGGRQQQQQGEAAPTPPRKRVYVCPEPTCV
Seita.9G24 LQLHRRGHNLPWKLRQRS---LPSG-----AGG----RQGDAAAAPPRKRVYVCPEPTCV
Sevir.9G24 LQLHRRGHNLPWKLRQRS---LPSG-----AGG----RQGDAAAAPPRKRVYVCPEPTCV
Bradi3g269 LQLHRRGHNLPWKLRQRSSSLQAPHSKQLGLG-------LGGGGDGPRKRVYVCPEPSCV
LOC_Os10g2 LQLHRRGHNLPWKLRHRAAAVSA----------------VTTAAPAPRKRVYVCPEPTCV
           ***************:*:    .                       ***********:**
                    sbid1-2 - 15M2-0483 - C->T - Q199*
Sobic.001G HHDPARALGDLTGIKKHFSRKHGEKRWRCERCGKRYAVQSDWKAHVKGCGTREYRCDCGI
GRMZM2G011 HHDPARALGDLTGIKKHFSRKHGEKRWCCERCGKRYAVQSDWKAHVKGCGTREYRCDCGI
Seita.9G24 HHDPARALGDLTGIKKHFSRKHGEKRWRCERCGKRYAVQSDWKAHVKGCGTREYRCDCGI
Sevir.9G24 HHDPARALGDLTGIKKHFSRKHGEKRWRCERCGKRYAVQSDWKAHVKGCGTREYRCDCGI
Bradi3g269 HHDPARALGDLTGIKKHFSRKHGEKRWKCERCGKRYAVHSDWKAHVKNCGTREYRCDCGI
LOC_Os10g2 HHDPARALGDLTGIKKHFSRKHGEKRWRCERCGKRYAVHSDWKAHVKNCGTREYRCDCGI
           *************************** **********:********.************

Sobic.001G LFSRKDSLLTHRAFCDALAEESARLLAAANNSSTISITTT------TSSSN--------N
GRMZM2G011 LFSRKDSLLTHRAFCDALAEESARLLAAAANNGS-TITTT------SSSNN--------N
Seita.9G24 LFTRKDSLLTHRAFCDALAEESARILAAANNGSTITTTNS------SNGGN------SGS
Sevir.9G24 LFTRKDSLLTHRAFCDALAEESARILAAANNGSTITTTNS------SNGGN------SGS
Bradi3g269 LFSRKDSLLTHRAFCDALAEESARILAAANNSST-----SNNSNI-INNNNNSVG--SSS
LOC_Os10g2 LFSRKDSLLTHRAFCDALAEESARLLAAANNSSSITTTTCNNSNISSNNNNNNINSISNS
           **:*********************:**** *..:             ...*        .

Sobic.001G DLL--N-TNNIAPLFLPFSN---SPP--VVAAQQNPNNTLFF--------LHQE---LSP
GRMZM2G011 DLL--NASNNITPLFLPFAS---SPPPVVVAAAQNPNNTLFF--------LHQE---LSP
Seita.9G24 DLLFSNSSGVVTPLFLPFPNPPPSPPPPAAAAAQNPNAFYFL--------HHQEQQLAAP
Sevir.9G24 DLLFSNSSGVVTPLFLPFPNPPPSPPPPAAAAAQNPNAFYFL--------HHQEQQLAAP
Bradi3g269 NNLL-----SPPPALFPTFS----------SHAQNPNPLMFLSEETHYHHHHQQEQ-LLP
LOC_Os10g2 NNLLITSSSSSPPLFLPFST---TP-----AENPNPNQLLFL--QQHQAAHHQL---LLP
           : *        .* ::*  .          :   ***   *:         **      *

Sobic.001G FLQPRMM-MQQPSPYLDLHMH--VDASITTTTTT------GGG----GILTDSPVLSFGL
GRMZM2G011 FLQPRVTMQQQPSPYLDLHMH--VDASIVTTT----------G----GLADGTP-VSFGL
Seita.9G24 FLHPRMV---QPSPYLDLH----ADAAAVTTATC--------G----GIGADT--VNFGL
Sevir.9G24 FLHPRMV---QPSPYLDLH----ADAAAVTTATC--------G----GIGADT--VNFGL
Bradi3g269 PFQPQYS-------YLS-HVDLPISSAITSGVSAFDTDR------------DT--LSFGL
LOC_Os10g2 QFQQPPS---SPPAYFD-HLAFGGGGGVITGSSCNDDNSSIAGDVMVAAGGDS--VSFGL
            ::           *:. *     ...  :                     .:  :.***

Sobic.001G ATDGSVAT----VGHRRLTRDFLGVDGGGHQVEELP--QLP--LCATAAA--------AS
GRMZM2G011 ALDGSVAT----VGHRRLTRDFLGVDGGGRQVEEL---QLP--LCATAAA------AGAS
Seita.9G24 APDGSVA-----LRHRRLTRDFLGVDGGS-QVEEL---QLP--IYATAAA------AAAT
Sevir.9G24 APDGSVA-----LRHRRLTRDFLGVDGGS-QVEEL---QLP--IYATAAA------AAAT
Bradi3g269 TPEGSVTMHA--GGRRGLTRDFLG----AGEVVEDQL-QMP--LCSAAAY------QGRP
LOC_Os10g2 TSEGSVTMHAGDVGRRRLTRDFLGVDHDAGEVDELELDELPADLSTTAAACQGCNFAAAT
           : :***:       :* *******    . :* *    ::*  : ::**          .

Sobic.001G ---RTASCAT-DLT--ARQYLGGR-LPPVNETWSHNF
GRMZM2G011 ---RTASCAT-DLT---RQCLGGR-LPPVNETWSHNF
Seita.9G24 VVPRAASCAT-DLT---RQYLGERPLPPVNETWSHNF
Sevir.9G24 VVPRAASCAT-DLT---RQYLGERPLPPVNETWSHNF
Bradi3g269 VA-TAVCCATTDLT---RQYLG-R-LPPVSETWSHHF
LOC_Os10g2 ---TAACCAT-DFTTGSRQYLG-R-LPPVNETWSHNF
               :..*** *:*   ** ** * ****.*****:*
```

SFigure 3. Alignment of Id1 homologs from C4 and C3 grasses. CLUSTAL format alignment by MAFFT (v7.511) (2) of Id1 proteins *Sorghum bicolor*: Sobic.001G242900 (Sobic.001G), *Zea mays*: GRMZM2G011357 (GRMZM2G011), *Setaria italica*: Seita.9G247600 (Seita.9G24), *Seteria viridis*: Sevir.9G247100 (Sevir.9G24), *Brachypodium distachyon*: Bradi3g26910 (Bradi3g269), and *Oryza sativa*: LOC_Os10g28330 (LOC_Os10g2). Blue highlighting indicates conserved Cys2His2 zinc finger domain residues. Bold and underlined text indicate *sbid1-1* and *sbid1-2* EMS mutation location, nucleotide change, name of original EMS line, and consequences for SbId1 protein.

SUPPLEMENTAL REFERENCES

1. Wang L, Lu Z, Regulski M, Jiao Y, Chen J, Ware D, et al. BSAseq: an interactive and integrated web-based workflow for identification of causal mutations in bulked F2 populations. Bioinformatics. 2021;37(3):382-7.

2. Katoh K, Kuma K-i, Toh H, Miyata T. MAFFT version 5: improvement in accuracy of multiple sequence alignment. Nucl Acids Res. 2005;33(2):511-8.
